# A Supervised Deep Learning Model Was Developed to Classify Nelore Cattle (*Bos indicus*) with Heat Stress in the Brazilian Amazon

**DOI:** 10.3390/ani16020161

**Published:** 2026-01-06

**Authors:** Welligton Conceição da Silva, Jamile Andréa Rodrigues da Silva, Lucietta Guerreiro Martorano, Éder Bruno Rebelo da Silva, Cláudio Vieira de Araújo, Raimundo Nonato Colares Camargo-Júnior, Kedson Alessandri Lobo Neves, Tatiane Silva Belo, Leonel António Joaquim, Thomaz Cyro Guimarães de Carvalho Rodrigues, André Guimarães Maciel e Silva, José de Brito Lourenço-Júnior

**Affiliations:** 1 Postgraduate Program in Animal Science (PPGCAN), Institute of Veterinary Medicine, Federal University of Para (UFPA), Federal Rural University of the Amazon (UFRA), Brazilian Agricultural Research Corporation (EMBRAPA), Castanhal 68746-360, PA, Brazil; eder.b.rebelo@gmail.com (É.B.R.d.S.); camargojunior@gmail.com (R.N.C.C.-J.); tatianebelovet@gmail.com (T.S.B.); jleokim.a@hotmail.com (L.A.J.); thomazguimaraes@yahoo.com.br (T.C.G.d.C.R.); andregms@gmail.com (A.G.M.e.S.); joselourencojr@yahoo.com.br (J.d.B.L.-J.); 2 Institute of Animal Health and Production, Federal Rural University of the Amazon (UFRA), Belem 66077-830, PA, Brazil; jamileandrea@yahoo.com.br; 3 Embrapa Eastern Amazon, Santarem 68010-180, PA, Brazil; lucieta.martorano@embrapa.br; 4 Department of Agricultural and Environmental Sciences, Federal University of Mato Grosso (UFMT), Sinop 78550-728, MT, Brazil; cvaufmt@gmail.com; 5 Institute of Animal Science, Federal University of Western Para (UFOPA), Santarem 68040-255, PA, Brazil; kedson_neves@hotmail.com

**Keywords:** air temperature, neural network, rectal temperature, respiratory rate

## Abstract

Non-invasive and intelligent technologies have been utilized to monitor agricultural systems in real time, facilitating expedient decision-making and the reduction in animal stress in diverse climatic conditions. The objective of this study was to develop a deep learning supervised model to classify Nelore cattle (*Bos indicus*) into two groups: those in comfort and those under thermal stress. Thirty cattle, aged between 18 and 20 months, were evaluated between June and December 2023, resulting in 676 samples collected across four daily periods (6:00, 12:00, 18:00, and 24:00). Biotic variables included rectal temperature (RT) and respiratory rate (RR), while abiotic variables included air temperature (AT) and relative humidity (RH). The neural network model exhibited an accuracy and recall of 72% but a low specificity of 42%. These metrics indicate that while the model is effective in detecting stress situations, it faces challenges in correctly identifying animals in thermal comfort, likely due to class imbalance and the need for additional input features to capture environmental adaptability. Consequently, it can be posited that supervised learning models are valuable tools for precision livestock farming, provided that discriminatory limitations are mitigated by refining input characteristics and data balancing.

## Simple Summary

Intelligent technologies that do not require the handling of animals have been used to monitor agricultural production systems in real time using captured images, enabling efficient decision-making and, consequently, minimizing animal stress. In this way, we used a deep learning model to classify Nelore cattle (*Bos indicus*) into two groups (comfortable and above thermal comfort) based on data collection at four different times, from June to December 2023, considering abiotic and biotic variables. After analysis, it was possible to see that the model used showed excellent accuracy, high precision and recall but low specificity in animals above comfort. Canonical analysis indicated that rectal temperature (RT) was a reliable predictor variable. Thus, it is concluded that the model has potential, but it is recommended that more variables be adopted to improve its classification capacity.

## 1. Introduction

Global warming has a direct influence on climate change, bringing with it several concerns, including threats to sustainability and species survival [1,2]. Extreme increases in air temperature (AT) and relative humidity (RH) contribute to the prevalence of heat stress in cattle, particularly in tropical and subtropical regions [3]. The demand for livestock products is growing, but the adverse effects of climate change caused by global warming directly affect productivity, resulting in economic losses for livestock farmers [4,5]. When animals are subjected to excessive heat and are unable to perform the thermoregulation process, a phenomenon known as heat stress is triggered [6,7]. This results in adverse effects on animal physiology due to the extreme temperatures experienced [8]. The effects of heat exposure can alter the conditions of nutrition, production (in dairy or beef cattle), health, development, and immunity, resulting in economic losses for the producer [9,10]. Other physiological issues include decreased milk production [11], panting, elevated body temperature [12,13,14], reduced feed consumption [15], impaired feed conversion rate, and increased respiratory rate [16,17,18].

The assessment of the welfare of grazing animals and the effects of climate variables on animals by researchers is not a recent practice. However, it has increasingly been the subject of scientific research aimed at quantifying the harmful effects and physiological limits of animals [19,20]. Conversely, the concern for animal welfare is a relatively recent phenomenon among livestock farmers, largely due to the negative impacts on productivity. Among consumers, the use of practices that promote the comfort of these animals has also been a point of concern [21]. Heat stress affects the intake and digestibility of ruminants, and since the animal tends to reduce grazing and rumination behavior in an attempt to avoid the production of metabolic heat, it can also cause an increase in body temperature, altering parameters such as heartbeat due to vasodilation, respiration rate, and reproduction of these animals, even resulting in death from heat stress [22,23].

In environments with intense solar radiation and high levels of AT and RH [24,25], animals engage in physiological processes mediated by the hypothalamus, including increased peripheral circulation, vasodilation, and sweating, to dissipate the absorbed heat. In cattle, body temperature can vary between 38.0 and 39.3 °C, thus necessitating the use of different forms of evaluation. These include rectal temperature, vaginal and oral temperature, and surface temperature (head, neck and croup) through infrared thermal imaging [26]. The choice of method is dependent on the animal’s response, invasiveness, and the precision of the chosen method [27,28]. In this context, the application of technologies in precision livestock farming represents a strategy for enhancing production efficiency in the face of climate change, with the objective of mitigating the adverse effects on animals. Consequently, the deployment of sophisticated technologies such as deep learning (DL) is intended to address challenges through intricate algorithms, facilitating the transfer of data to networks of interconnected neurons or neural networks. In the context of livestock farming, the application of DL can facilitate the identification of issues pertaining to animal health, management, nutrition, and welfare [29].

The application of novel technologies for the assessment of thermal comfort in grazing cattle offers promising and innovative approaches based on DL, thereby substantiating the necessity for the utilization of these methodologies in livestock farming, particularly in the Amazon, which is characterized by climatic conditions that are highly distinctive of tropical regions, including elevated temperatures and humidity, as well as intense solar radiation, which has a detrimental impact on the health and well-being of these animals [30]. The conventional methodologies employed to categorize the thermal comfort of cattle entail the utilization of relatively simplistic indices that frequently neglect to encompass the intricate interactions between abiotic and biotic variables, along with their impact on animal comfort [31].

Prior research has illustrated the deployment of DL in property management, livestock farming, with an emphasis on environmental sustainability, and precision livestock farming [32]. Conversely, the advancements observed in DL have the potential to capture the weights of information present in features, offering a distinct advantage over other methods due to its inherent learning process. This enables the updating of new weights related to features, thereby enhancing classification performance and increasing accuracy [33]. Furthermore, the utilization of non-invasive and intelligent technologies to monitor the various breeding systems with immediate responses through the capture of images in real time enables more expedient decision-making, thereby reducing the stress experienced by the animals in diverse climatic conditions [34].

It is evident that supervised machine learning tools, and, in particular, DL tools, offer significant advantages. One such advantage is the ability to establish information relationships between classificatory variables without imposing specific and adjacent assumptions on the distribution of sample data [28]. Nevertheless, given the growing interest in these techniques across various animal science applications and the promising potential of using classification models to assess thermal comfort in beef cattle in the Amazon region, incorporating biotic and abiotic variables [30], further investigation is warranted. In this context, it is postulated that the implementation of a multilayer artificial neural network model, which is deemed an appropriate and highly promising approach for classifying Nelore cattle into thermal comfort ranges, is feasible. This classification is based on the animals’ RR, AT, and RH, which serve as classifying variables and features in the model.

The aim of this study was to develop a supervised deep learning model for the classification of Nelore cattle (*Bos indicus*) into two groups: those in comfort and that above thermal comfort. In this study, deep learning was chosen for its ability to capture nonlinear relationships and complex interactions between environmental and physiological variables, which are common in thermophysiological responses in cattle.

## 2. Materials and Methods

### 2.1. Ethics and Location

All experimental procedures involving animals were approved by the Animal Use Ethics Committee in May 2023, with protocol number CEUA-UNAMA 0001-87/2023.

The municipality of Mojuí dos Campos, located in the northern region of the state of Pará, Brazil, served as the setting for this study, specifically on a farm where beef cattle are bred, raised, and fattened. The region is characterized by a hot and humid climate of the Am4 type, with total precipitation reaching 60 mm during the less rainy periods and an annual precipitation range of 1900–2100 mm. The average air temperature is 25.6 °C, with relative humidity of 84% and 86% [35].

In this context, Martorano et al. [36] propose that this is a transitional climatic condition with Aw3 (which refers to A, the tropical climate; W, the dry season; and 3, the details of the climatic patterns) in the Brazilian Amazon. The period from February to April is considered the least rainy, while the period from August to October is the rainiest [36].

### 2.2. Weather Data

**Figure 1:**
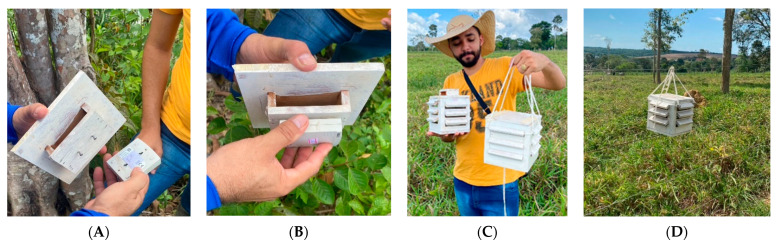
Installation of agrometeorological sensors. (**A**) The agrometeorological box in an open position, with the sensor in hand. (**B**) The sensor fitted to the interior of the agrometeorological box. (**C**) The box assembled with the sensor, and (**D**) the agrometeorological box installed on the fence that forms the experiment paddock.

An agrometeorological station (Figure 1A–D) (HOBOWARE Onset H21 002) was previously installed on the property in Mojuí dos Campos, Pará, specifically in the paddock where the animals were kept. This station provided daily data on air temperature (AT °C) and relative humidity (RH %) during the months of June to December 2023. The agrometeorological station was set up in accordance with the recommendations of the World Meteorological Organization (WMO).

### 2.3. Animals, Management and Breeding Systems

In this study, a total of 30 animals were randomly selected from a population of cattle, all of which were male, uncastrated, with similar coat color and of the Nelore breed. The average weight was 250 ± 36 kg, with a body condition score (BCS) of 3.0 and an age between 18 and 20 months. The sample size (*n*= 30) and group uniformity were strategically chosen to minimize biological noise and prioritize the environment–physiology interaction, following established standards for intensive physiological studies with repeated measures. This age range was selected due to the availability of animals on the farm and the intention of observing them for six months, during which time they would remain in these systems. In total, nine paddocks were made available, with each breeding system having 3 paddocks, so that the animals could be rotated every 28 days. To divide the systems, the animals were divided into three groups of 10, corresponding to the types of breeding systems: silvopastoral, traditional and integrated.

**Figure 2:**
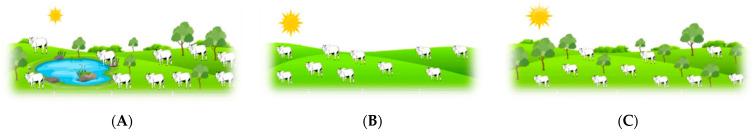
Experimental groups. (**A**) Integrated System (IS), which had trees providing shade, water for bathing and drinking, and mineral salt; (**B**) Traditional System (TS), which had no trees or shade, but included access to water for drinking and mineral salt; (**C**) Silvopastoral System (SP), which included trees providing shade and access to water and mineral salt.

The experimental groups were as follows (see Figure 2A–C):

- Silvopastoral System (SP)—which included trees providing shade and access to water and mineral salt;
- Traditional System (TS)—which did not have trees or shade but did include access to water and mineral salt;
- Integrated System (IS)—which had trees providing shade, water for bathing and drinking, and mineral salt.

All paddocks contained *Brachiaria brizantha* cv. Marandú pasture, with an area of 1.7 hectares each, resulting in a total area of 15.3 hectares. The animals were fed basically on pasture and mineral salt ad libitum.

The animals were initially subjected to an adaptation period of seven consecutive days, after which they were gradually introduced to the experimental management procedures, including the handling of the restraint log for the collection of physiological parameters. The animals were led at a slow pace on foot to the pen and then placed in the log appropriately, without the use of stressful stimuli. To ensure data reliability and minimize inter-observer bias, all measurements were conducted by a single trained observer following a standardized protocol every 28 days (four times daily). The data obtained between June and December were submitted to the intelligent system, resulting in a total of 676 observations for the entire study for each physiological parameter evaluated.

### 2.4. Collection of Respiratory Rate and Rectal Temperature

The RR and RT of the cattle were collected at four different times: 06:00, 12:00, 18:00, and 24:00. These times were selected to align with the months of June to December 2023. Data collection occurred every 28 days, resulting in a total of 676 observations for each physiological variable throughout the entire study. All assessments were conducted by a single trained observer to ensure data reliability and minimize inter-observer bias. The RR was obtained through a macroscopic inspection of the thoraco-abdominal movements of the cattle. Consequently, a digital stopwatch was utilized to ensure that each evaluation was conducted for precisely 60 s. The RT was obtained via transrectal administration of a veterinary clinical thermometer (Model 5198.10, Incoterm, São Paulo, Brazil), which had a maximum range of 44 °C, in accordance with the recommendations of Dirksen et al. [37].

### 2.5. Benezra Comfort Index (BTCI)

The Benezra index is calculated according to the following formula:

BTCI = RT/38.33 + RR/23

where RT = rectal temperature (°C) and RR = respiratory rate in respiratory movements per minute (rmm).

BTCI values exceeding 2.0 indicate thermal discomfort due to the animals’ inability to adapt to their surrounding environment. Conversely, BTCI values below 2.0 suggest a greater capacity for adaptation.

### 2.6. Method of Classification of Groups

In this study, RT was utilized as the primary criterion for classifying the animals, as it is a more stable and reliable metric [17,35]. The normal range for RT is 38.0 °C to 39.3 °C. Accordingly, the animals were divided into two categories.

- In thermal comfort: composed of animals that reached a maximum of 39.3 °C.
- Above thermal comfort: composed of animals that have exceeded this RT threshold.

### 2.7. Model Architecture in Deep Learning

The DL model was constructed using a neural network based on the Multilayer Perceptron (NNAMP) architecture, employing a feed-forward type and the backpropagation training algorithm. The choice of this architecture was driven by its ability to model complex non-linear interactions between environmental and physiological variables, a characteristic where linear and traditional tree-based models may face limitations in animal bioclimatology. Furthermore, the MLP was preferred for its capacity to establish informational relationships without strict assumptions regarding sample data distribution, utilizing the BTCI as a biological baseline for performance comparison.

Its objective was to categorize the data into two distinct groups: those indicating comfort and those indicating superior thermal comfort. The backpropagation algorithm was selected for use in this study because it is a standard method for training artificial neural networks. This approach allows the gradient of a loss function to be calculated in relation to all the weights in the network. This enables the weights of the characteristics to be adjusted in a manner that reduces error rates and improves the reliability and accuracy of the model. In the NNAMP architecture, the number of features that fed the input layer was equivalent to the effects of the independent variables. The variables under consideration were RT, RR, AT, and RH (four input variables).

Accordingly, an array of hidden layer configurations was evaluated, including variations in the number of neurons per layer. The initial testing of the neural network involved a single hidden layer with two neurons, representing the input of four features (RT, RR, AT, RH) and the output of one. Subsequently, the number of neurons was augmented until no discernible improvement in accuracy was observed. The most efficient architectural configuration, composed of an input layer with four neurons (one for each input feature) and two hidden layers with six neurons each, yielded the highest accuracy.

To mitigate the issue of overfitting during training, dropout layers with an intensity of 20% were incorporated into the artificial neural network architecture and interspersed between the layers. The dropout technique entails the deliberate omission of neurons that serve as feature detectors within the neural network during each stage of training. The exclusion of each neuron is determined randomly, thereby minimizing the effect of overfitting. This is achieved because each neuron becomes independently sufficient, in the sense that the neurons within the layers learn weight values that are not based on the cooperation of their neighboring neurons. The influence of varying percentages (10, 20, 30, and 40%) was examined. It was observed that from 20% onwards, there were no discernible changes in the accuracy metric.

The activation function employed in the input layers and hidden layers was ReLU (Rectified Linear Unit), whereas in the output layer, the sigmoid activation function was utilized. In the construction of the model, the stochastic descending gradient learning optimization function (Adam) was employed, with a batch size of 16 and a maximum number of iterations of 1000. Batch sizes of 4, 8, 12, 16, 20, and 22 were tested, and the results indicated that a batch size of 16 yielded the most accurate results. Despite imposing a maximum of 1000 iterations, the network exhibited convergence at an earlier point in time. This was merely a precautionary measure in the event that the values obtained for the variance in the gradient descent function remained constant and resulted in a time-consuming computational process.

The NNAMP was constructed using the Python programming language [38] (version 3.13), specifically with the Spyder and the Keras library, which is a TensorFlow. The efficacy of the NNAMP was assessed through the use of a confusion matrix.

The total sample was divided into two distinct subsets: a training sample and a test sample (validation). The training sample constituted 80% of the total sample size, while the test sample accounted for the remaining 20%. The features were standardized using the StandardScaler function of the scikit-learn preprocessing library (version 0.24.2).

**Figure 3:**
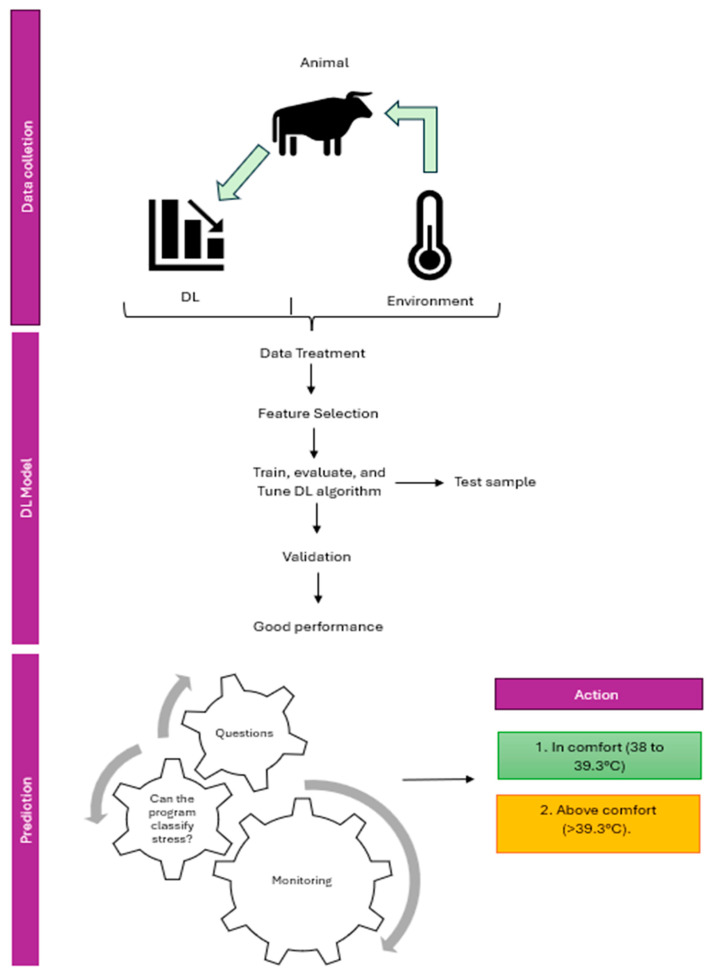
Detailed diagram of the information describing the deep learning model used during the preparation and execution of the analysis to classify cattle. DL = deep learning.

Figure 3 illustrates the diagram containing the DL model description information during the preparation and execution of the analysis. This information can be utilized to assist the farmer in classifying the animals and subsequently implementing measures to mitigate the adverse effects of stress on the animal. The receiver operating characteristic (ROC) curve was employed for the assessment of the model’s performance. Information regarding the RT of the animals was employed to categorize them into two distinct classes: Class 1, comprising animals with RT below 39.3 degrees Celsius, and Class 2, comprising animals with RT equal to or greater than 39.3 degrees Celsius. This constituted the structured data essential for supervised machine learning. Accordingly, the DL model employed data from the animals’ respiratory rate records, in addition to air temperature and relative humidity records, as input variables. The total sample was divided into a training set and a validation set, and the quality of the model was assessed based on the accuracy, precision, recall, and F1-score metrics obtained by the model in the validation set.

The model was trained by minimizing the binary cross-entropy loss function. The weights were updated using the Adam optimization algorithm, with a learning rate of 0.01, a value selected automatically during the hyperparameter optimization process. The final choice of hyperparameters was based on maximizing predictive performance in the validation set, ensuring an adequate balance between model fit and generalization, in which different configurations were evaluated based on performance in the validation set, and the model with the best accuracy was selected. The final model selected has the following configuration: Input layer composed of three continuous predictor variables, previously standardized; first fully connected hidden layer, containing 32 neurons and ReLU activation function, followed by a Dropout layer with a rate of 0.20; second fully connected hidden layer with 20 neurons and ReLU activation function, followed by Dropout of 0.30; third fully connected hidden layer with 4 neurons and ReLU activation function; and output layer formed by one neuron, using the sigmoid activation function, appropriate for binary classification problems. The resulting neural network has 877 trainable parameters, reflecting a compact structure appropriate for the size of the dataset, reducing the risk of overfitting.

### 2.8. Canonical Correlation

In order to gain insight into the relationship between the neural network input variables (features) and the RT of the animals, as well as the impact of this relationship on the performance of the neural network, a canonical correlation analysis was conducted. RT was a reliable predictor because it is physiologically more stable than respiratory rate. Rectal temperature directly reflects the animal’s core heat balance and is less influenced by transient stimuli such as handling, momentary physical activity, or excitement. In contrast, respiratory rate is a rapid and adaptive response to thermal stress, highly variable and sensitive to immediate factors, which increases its variability and reduces its stability as an isolated predictor. Furthermore, the Nelore breed is highly adapted to high temperatures, such as those found in the tropics.

This analysis considered two groups of variables: biotic variables (RT and RR) and abiotic variables (AT and RH). The canonical correlation analysis was conducted using the eigenvectors associated with the variables employed, with the objective of assessing the degree of covariance between and among the groups of biotic and abiotic variables. This approach was adopted in order to facilitate the interpretation of the neural network evaluation, given that when the correlations between variables are low, the performance of any prediction model is also diminished. It is postulated that the canonical correlation technique is more informative than a Pearson correlation matrix, as it allows for the detection of the contribution of each eigenvalue associated with each eigenvector.

## 3. Results

The descriptive statistics of the parameters of biotic and abiotic variables in each class are presented in Table 1.

Table 2 presents the descriptive statistics for the predictor variables in the two samples. It can be seen that the random process of sample formation, testing, and training resulted in the emergence of highly similar parameters in both samples.

The performance of the classification model in relation to its different discrimination thresholds can be evaluated by observing the Receiver Operating Characteristic (ROC) curve in Figure 3. In light of the aforementioned classification of animals in thermal comfort, it is possible to posit that those exhibiting positive characteristics could be considered as such, whereas those displaying characteristics above comfort could be regarded as negative. The graphical representation thus depicts the proportion of values classified as negative that are incorrectly classified as positive on the abscissa axis (false positive rate). The false positive rate (FPR) is calculated as follows: FPR = FP/(FP + TN), where FP represents the number of false positives and TN denotes the number of true negatives. Conversely, the ordinate axis represents the true positive rate (TPR), which is the proportion of positive examples that are correctly classified as such. The TPR is calculated as TPR = TP/(TP + FN), where TP is the number of true positives and FN is the number of false negatives.

The random diagonal (dotted line) represents the expected performance of a random classifier. In the case of a random classifier, it is expected that the true positive rate will be equal to the false positive rate, resulting in a ROC curve along this diagonal dotted line. In such a case, the classification model would be deemed inadequate.

The area under the ROC curve (AUC) is a measure of the model’s capacity to discriminate. A higher AUC value indicates superior model performance. An AUC of 0.5 indicates a random performance, whereas an AUC of close to 1.0 indicates a perfect performance. In the evaluated model, the area corresponded to 0.67, indicating a good model performance.

**Figure 4:**
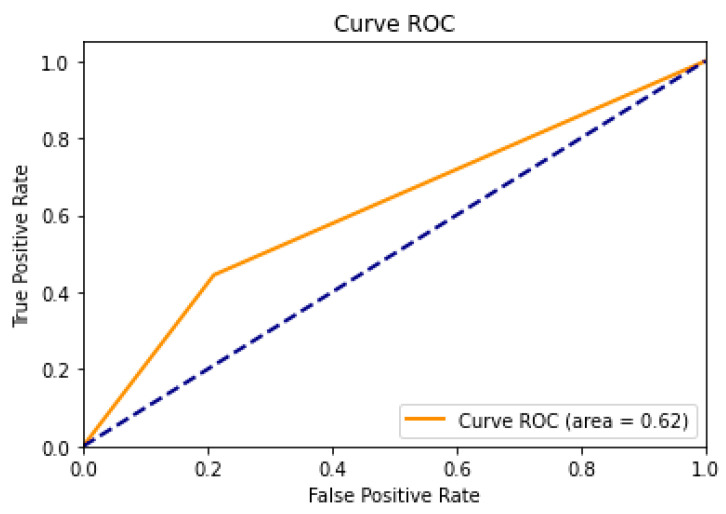
Receiver Operating Characteristic (ROC) curve obtained by the model in the test sample to evaluate the model performance.

The optimal operating point is defined as the point on the ROC curve that maximizes sensitivity and minimizes the false positive rate. This point represents the optimal equilibrium between accuracy and a graded error rate. In this scenario, the further the ROC curve deviates from the random diagonal towards the upper left quadrant of the graph, the superior the model’s performance, as illustrated in Figure 4.

The confusion matrix related to the model is presented in Table 3. The model demonstrated an accuracy of 0.72, calculated as the sum of the diagonal elements divided by the sum of all values. The model demonstrated an accuracy of approximately 80% in predicting the comfort class, with a recall of 83% for correctly identifying animals belonging to this class. Conversely, the specificity, defined as the proportion of animals classified by the model as belonging to the aforementioned thermal comfort class that was correctly identified, corresponded to 42%. The F1 score, which represents a harmonic mean of precision and recall, is calculated as follows: the F1 score, defined as the harmonic mean of precision and recall, was calculated to be 81.3%.

In conclusion, the high accuracy rate indicates that the model exhibits minimal error in classifying animals within the comfort class when they truly belong to that class. Similarly, the high recall rate suggests that the model also makes a few errors in classifying animals above comfort when they are, in fact, within the comfort class. The accuracy of the model was demonstrated by its ability to correctly classify 72% of all animals in the test sample. Nevertheless, the specificity was 42%. This suggests that the model demonstrated a relatively low level of accuracy in classifying the animals in the aforementioned thermal comfort category.

To enhance comprehension of the neural network’s performance, the outcomes of the canonical correlation analysis were examined. This analysis classified the variables into two groups: the first group encompassed the biotic variables (RT and RR), designated as BV, while the second group comprised the abiotic variables (AT and RH), designated as AV. The findings revealed that the BV group was the primary determinant of the neural network’s behavior.

The RT had a greater influence on the formation of BV and AT with AV (Table 4). The canonical correlations indicated that the most relevant variables were RT and AT, exhibiting a moderate–low correlation. These findings are consistent with those presented in Table 1, which showed that animals in the comfort class exhibited higher RT values and were above the higher AT values, as well as lower RH. Notably, there were no differences in RR between the two classes, indicating adaptability to unfavorable environmental conditions in two of the animals. Therefore, the input features utilized in the neural network were inadequate for differentiating the animals with regard to heat stress.

**Figure 5:**
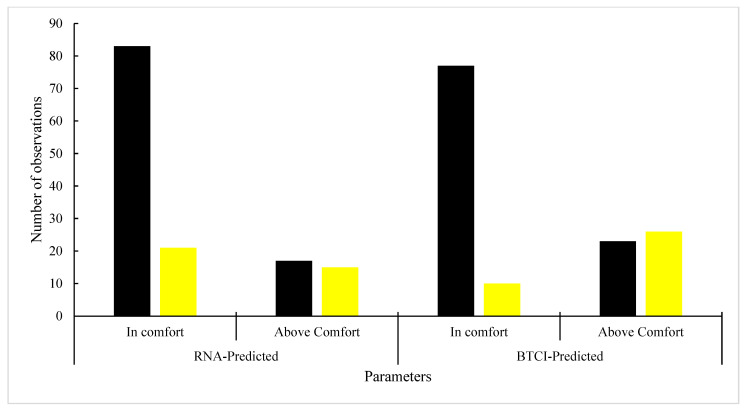
Comparison of metrics between values predicted by the ANN model and through the BTCI.

A comparison of the metrics between the values predicted by the artificial neural network (ANN) model and those derived from the BTCI, with the classification of the animals, revealed a high degree of similarity in the resulting accuracies (0.72 and 0.75), precision (0.80 and 0.88), recall (0.83 and 0.77), and F1-score (0.81 and 0.82) for the ANN model and the BTCI, respectively. These findings are illustrated in the confusion matrices presented in Figure 5.

A comparison of the confusion matrix (Table 5) between the classifications of the ANN model and the BTCI revealed that the accuracy, precision, recall, and F1-score metrics were, respectively, equal to 0.92, 0.99, 0.89, and 0.94. This confirms the similarity between the classification methods.

The innovation of this research lies in the application of deep neural networks specifically adapted for Nelore cattle, a breed recognized for its hardiness but which still requires precise monitoring to optimize performance under climate change scenarios. Unlike previous studies that utilize static models or focus exclusively on taurine breeds (*Bos taurus*), this work contributes by demonstrating the viability of dynamic learning systems that utilize easily obtainable abiotic and biotic variables. Furthermore, the integration of Canonical Correlation Analysis (CCA) with the ANN provides a crucial layer of biological interpretation, validating the importance of physiological variables in stress prediction—an advancement over traditional ‘black-box’ computational models.

However, it is important to note that both models share a moderate specificity (42%), indicating that while they are highly effective in detecting animals under thermal stress, they tend to generate false alarms for those in thermal comfort. This limitation suggests that the boundary between comfort and the onset of stress is complex and may require additional indicators for a clearer separation between classes. The principal advantage of employing ANN models is that the network can be continually restructured and updated, thereby sustaining a continuous learning process.

## 4. Discussion

The results obtained demonstrate the positive impact of welfare on livestock through the implementation of strategic initiatives and the optimization of the production process. The development of precise models and the implementation of novel technologies in livestock to assess thermal comfort enable livestock farmers to promptly and strategically identify interventions that consider climatic factors to enhance animal management and feeding, thereby impacting herd welfare and animal productivity.

The use of the model allows for interventions and decision-making by the farmer without the need for constant animal management, so the application of the model within rural properties will be important to enhance animal welfare and reduce the time spent during extensive data collection. Therefore, it can promote the efficient switching of animals between pastures or paddocks, as indicated by the model used in this study.

The test sample presented satisfactory parameters, demonstrating a performance of 72%, which indicates the effectiveness of the proposed model. Specifically, low indicates a higher rate of false positives, that is, animals classified as being above thermal comfort when, biologically, they are still in adequate conditions. This limitation suggests that the model tends to prioritize the detection of animals at risk, which may be desirable from an animal welfare perspective, but requires caution in interpreting the results and in the practical use of the model in breeding systems. These results are closely in line with those previously reported by Sousa et al. [39], who obtained 71% (R2 of 0.50) when comparing two groups of Nelore cattle using a neural model with feed-forward and multilayer architecture.

In the study by Sousa et al. [40], which also employed a two-group division of Nellore cattle, the fuzzy classifier yielded a model test performance of 81 (R2 of 0.74%). Both studies aimed to predict stress through the use of physiological variables, with the construction of prediction models.

The application of technologies in livestock farming facilitates the promotion of animal welfare and the assurance of the quality of animal products. This is achieved by providing producers with viable alternatives that can effectively mitigate and prevent the adverse effects of heat stress on animal health, which subsequently reduces losses incurred during the production process [41,42].

Accordingly, the application of the LD model in livestock farming entails the assessment of the influence of climatic variables on the comfort and, subsequently, on the well-being of animals, which is inextricably linked to economic losses and the quality of animal products [43]. The method is effective in identifying and classifying animals according to their level of comfort or heat stress. This enables farmers to implement prevention practices and adapt the environment in which animals are raised, thereby mitigating the impact of climate change on them. Furthermore, the utilization of the LD model exemplifies the capacity for innovation and sustainable adaptation of a property, taking into account the requirements of consumers and environmental resources, such as the promotion of animal well-being throughout the animal’s lifespan and the integration of forest areas within pastures [40].

In order to enhance comprehension of the neural network’s performance, the outcomes of the canonical correlation analysis, which incorporated the biotic variables (RT and RR) in one group and the abiotic variables (AT and RH) in another, revealed that the former group was the primary focus of the neural network.

The RT had a greater influence on the preparation of BV and AT with AV, as indicated by the canonical correlations, which identified RT and AT as the most relevant variables, although with a moderate–low correlation. These findings are consistent with those presented in Table 1, in which the animals in the comfort class exhibited higher RT values and were above the highest AT values, as well as lower RH. Notably, there were no differences in RR between the two classes, indicating that both animals demonstrated adaptability to unfavorable environmental conditions. Therefore, the input characteristics of the neural network were inadequate for differentiating between the animals based on their heat stress levels.

Therefore, the neural network model demonstrates good accuracy, high precision, and high recall but low specificity. This is attributed to the model’s difficulty in detecting animals with conditions above thermal comfort, which has the smallest number of animals and, consequently, less influence on the metrics inherent to the thermal comfort class. This limitation of the model can be attributed to the utilization of input variables (features) that offer minimal contribution to this discrimination.

The use of non-invasive methods to assess physiological conditions and the number of animals through LD makes it possible to associate climatic variables and physiological parameters. The periodic capture of data allows the algorithm to be trained to estimate heat stress indices, thus providing personalized information, the creation of alerts, solutions to mitigate heat stress, and data on the climate [44,45] automatically and in real time, as well as allowing the number of evaluations to be much greater, favoring consulting services and obtaining data for farms and also for the government [46].

The adaptability of cattle to their environment can be attributed to the lower thermal stress and, consequently, a higher level of comfort. This enables the activation of compensatory and adaptive mechanisms, such as changes in physiological functions, which seek to re-establish body homeostasis and, in turn, promote the well-being and survival of animals in hot environments [44].

Another factor that may have contributed is the development of acclimatization, which has been observed to trigger greater water intake in the animal, as well as a reduction in feed consumption and changes in physiological functions, including increased urination and defecation. The combination of these factors can significantly contribute to the majority of cattle being within the comfort zone [47].

The RT demonstrated a moderately low correlation with AT, which can be attributed to the tendency of animals to retain internal heat when exposed to high temperatures, leading to an increase in body temperature [48].

Furthermore, cattle may also exhibit reduced RT even in tropical climates, primarily due to their calm demeanor, robust development, and resilience to parasites [46]. Furthermore, the elevation in body temperature in cattle may be attributed to a multitude of factors, including climate, exercise and digestion, inflammation, and immune response [49]. Furthermore, alterations in the temperature gradient of +1 °C can occur within minutes of exposure, characterizing stress-induced hyperthermia (SIH) or emotional fever [44]. This phenomenon is frequently reported in diverse species, including cattle [50].

It was observed that RR remained unaltered even when AT was high, which coincided with the increase in RT. This may be indicative of the animals’ adaptability to their environment since it is known that RR is a variable used to evaluate stress in cattle [1]. The adaptability of cattle raised on pasture in tropical regions is of great importance, as their tolerance to high temperatures and relative humidity reduces the impact on productivity [51].

In light of these observations, the evolutionary process of animals, as exemplified by the Zebu cattle breed, has led to the development of thermotolerance in regions with more intense heat when compared to taurine breeds. This is attributed to the ability to exchange heat with the environment [30]. Conversely, taurine breeds also demonstrate considerable potential for adaptation to locations with higher temperatures through the process of natural selection [52].

The process of heat exchange that occurs through sweating represents one of the primary mechanisms of body temperature control, a function that is carried out cutaneously and in conjunction with the animal’s breathing. Therefore, when these processes ensure homeothermy, other forms of thermoregulation are not activated [53]. Consequently, the study did not observe any impact of RR, as it was not influenced by the breeding environment.

The findings of this study can be utilized in practical applications to estimate the heat load of animals in relation to their environmental context, employing LD. Furthermore, this model can be integrated into other studies to facilitate the management and nutrition of these animals [54]. Furthermore, the potential for utilizing this technology to facilitate the adaptation of animals to diverse environmental conditions on various livestock farms is noteworthy, making this an innovative technology within the domain of livestock farming [55,56].

One study used a heat stress monitoring model based on artificial intelligence in order to provide methods that would enable greater thermal comfort and showed that the models and processing units automatically activated heat stress reduction measures such as the use of water spray and environment modifiers. In addition, the authors show that the data obtained constitute a robust repertoire and that they can be stored and used in the future to observe the responses of the animals according to temperature variations, identifying the best-adapted animals [57,58] and the use of more favorable and effective production systems for these animals through the evaluation of physiological variables such as RT and RR, for instance.

The neural network model demonstrated satisfactory performance in classifying animals according to thermal comfort, as observed by Rodrigues et al. [59], when evaluating the method for extracting characteristics from infrared thermography data by applying the development of animal thermal stress classifier models. This class had the largest number of animals, and the model exhibited high precision and high recall, as well as good accuracy, based on the calculated metrics. However, the model demonstrated suboptimal performance in classifying the animals in the class above thermal comfort. This is likely due to the smaller number of animals in this class, which resulted in a lower metric value for specificity [60]. This outcome can be attributed to the limited correlation between the classification variables and the RT of the animals, given that the latter was employed for the categorization of the classes.

Other studies such as Brown-Brandl et al. [61], Hernandez-Julio et al. [62], and Pacheco et al. [63] evaluated the use of neural network models to predict thermal and physiological responses in cattle. The studies noted that the use of neural networks was able to capture complex responses between physiological and environmental variables.

One potential explanation for this low correlation between RT and the other model features is the adaptability of the animals to the conditions represented by the sampled rearing systems. Despite this, the animals in the thermal comfort class and those under thermal stress exhibited similar mean RR values, although there were notable differences between the classes in terms of RT, AT, and RH. However, these physiological nuances also highlight critical limitations of the current model.

The low specificity (42%) indicates a high rate of false positives, which is primarily attributed to class imbalance in the training data and the limited number of input features. Biologically, this outcome also reflects the ‘carry-over’ effect in Nelore cattle, where physiological responses such as RT and RR remain elevated for a period even after ambient heat stress diminishes, blurring the comfort-stress decision boundary. From a precision management perspective, this trend represents a conservative safety margin; the model prioritizes high sensitivity (94%) to ensure that heat stress events are not missed, which is more critical for preventive intervention in tropical systems than avoiding false alarms. To enhance environmental adaptability and discriminative power, future improvements should focus on including behavioral and physiological features, applying data balancing techniques, and testing the model across diverse climatic conditions.

## 5. Conclusions

This study demonstrates the effectiveness of deep learning as a technology for identifying thermal stress situations in cattle. While the classification model achieved high recall and precision, ensuring the reliable detection of critical heat stress, it exhibited a notably low specificity. This limitation indicates a significant rate of false alarms for the comfort class, likely stemming from class imbalance in the training dataset and the limited scope of the current input features. These findings suggest that variables such as RT, RR, and THI, while relevant, may not fully capture the biological complexity of environmental adaptability, highlighting the need for a more diverse set of metabolic and physiological indicators to enhance the model’s discriminative power.

Despite these performance constraints, the integration of artificial intelligence into animal monitoring remains a highly valuable tool for improving health management and animal welfare. The application of neural networks provides a robust framework for identifying metabolic profiles and thermal resilience, supporting more precise nutritional and genetic selection in livestock breeding. Future research should focus on balancing datasets and incorporating additional behavioral and environmental variables to improve model robustness and reduce false positive rates. Ultimately, this methodology offers a promising path for the continuous monitoring of animal–environment interactions, facilitating more sustainable and adaptive management practices.

## Figures and Tables

**Table 1:** Average of the data analyzed for RT, RR, AT and RH considering the standard deviation, lower (Lm.) and upper (Um.) values of the average confidence interval and sample size (*n*) for the thermal comfort classes.

	In Thermal Comfort (*n* = 452)			
	Average	SD	Lm.	Um.
Rectal Temperature (°C)	38.75	0.33	38.72	38.79
Respiratory Rate (mpm)	31.35	6.52	30.75	31.95
Air temperature (°C)	27.75	3.42	27.43	28.06
Relative Humidity (%)	59.95	15.84	58.48	61.41
	**Above Thermal Comfort (*n* = 224)**			
	**Average**	**SD**	**Lm.**	**Um.**
Rectal Temperature (°C)	39.56	0.20	39.53	39.59
Respiratory Rate (mpm)	31.86	6.80	30.96	32.76
Air temperature (°C)	29.35	2.83	28.98	29.72
Relative Humidity (%)	55.56	16.23	53.42	57.70

Note: SD = standard deviation. Lm = lower mean. Um = upper mean.

**Table 2:** Estimates of mean, standard deviation and sample size (*n*) for RT, RR, AT and RH, in the training samples, test samples and in the total sample.

	Training Sample (*n* = 540)			
Criminators	Average	SD	Min.	Max.
Respiratory Rate (mpm)	31.76	6.54	20.00	60.00
Air Temperature (°C)	28.31	3.26	22.10	34.00
Relative Humidity (%)	58.22	16.27	30.00	84.00
	**Test Sample (*n* = 136)**			
	**Average**	**SD**	**Min.**	**Max.**
Respiratory Rate (mpm)	30.57	6.84	16.00	56.00
Air Temperature (°C)	28.16	3.56	22.10	34.00
Relative Humidity (%)	59.59	15.39	32.00	84.00
	**Total Sample Size (*n* = 676)**			
	**Average**	**SD**	**Min.**	**Max.**
Respiratory Rate (mpm)	31.52	6.62	16.00	60.00
Air Temperature (°C)	28.28	3.32	22.10	34.00
Relative Humidity (%)	58.49	16.09	30.00	84.00

Note: SD = standard deviation. Min = minimum. Max = maximum.

**Table 3:** Confusion matrix for the classification of the animals obtained by the model in relation to the actual classification, in the comfort and above thermal comfort classes.

Real	Predicted	
	In Comfort	Above Comfort
In Comfort	83	17
Above Comfort	21	15

**Table 4:** Canonical coefficients between biotic (BV) and abiotic (AV) variables, with canonical correlations between the original and canonical variables (VA and VB).

Discriminators	Standardized Coefficient of the First	
	Canonical Pair	
Biotic Variables	BV	
Rectal Temperature (°C)	0.9503	
Respiratory Rate (mpm)	0.2295	
Abiotic Variables	AV	
Air Temperature (°C)	1.0796	
Relative Humidity (%)	0.1888	
Canonical correlation		
Biotic Variables	BV	AV
Rectal Temperature (°C)	0.97	0.34
Respiratory Rate (mpm)	0.32	0.11
Abiotic Variables	AV	BV
Air Temperature (°C)	0.98	0.35
Relative Humidity (%)	−0.34	−0.12

Note: BV = biotic variables, AV = abiotic variables.

**Table 5:** Comparison of the confusion matrix between the classifications of the ANN model with the BTCI.

ANN Predicted	BTCI Predicted	
	In Comfort	Above Comfort
In Comfort	86	10
Above Comfort	1	39

Note: ANN = Artificial Neural Net; BTCI = Benezra Comfort Index.

## Data Availability

The data presented in this study are available upon reasonable request from the corresponding author.
